# Dual-band composite high gain MIMO antenna for 5G NR applications employing shareable small cell radio unit

**DOI:** 10.1038/s41598-026-39955-w

**Published:** 2026-02-15

**Authors:** Asadullah, Nosherwan Shoaib, Muhammad U. Khan, Ali Ahmed, Atef A. Aburas, Qammer H. Abbasi

**Affiliations:** 1 School of Electrical Engineering and Computer Science (SEECS), National University of Sciences and Technology (NUST), Islamabad, 44000 Pakistan; 2 Telecom R&D Department, Advanced Communications & Electronics Systems (ACES), Riyadh, Kingdom of Saudi Arabia; 3 School of Engineering, James Watt Building (South), University of Glasgow, Room 626, University Avenue, Glasgow, G12 8QQ UK; 4 College of Engineering, Abu Dhabi University, Abu Dhabi, United Arab Emirates

**Keywords:** Engineering, Physics

## Abstract

In this paper, a MIMO antenna is presented consisting of 4 composite elements. Each antenna element consists of a U-shaped conducting structure and a perturbed barrel Dielectric Resonator Antenna (PB-DRA) structure. The former is loaded with a bow-tie patch, and it is parasitically excited through the U-shaped microstrip element. The additional bow-tie structure is designed for the gain enhancement in the 5G NR frequency range 1 (FR1) band and frequency range 2 (FR2). The PB-DRA has a dielectric constant of 8, and it is excited in higher-order mode to achieve resonance in FR2. The composite structure offers dual-band resonance with 3.85 GHz resonant frequency in the FR1 band and 26.65 GHz in the FR2 band, giving a large frequency ratio radiation characteristic. The proposed antenna offers impedance bandwidth of 2.02 GHz in the FR1 band, and 5.3 GHz in the FR2 band. Achieving multi-band resonance with a large frequency ratio is essential for exploiting the true benefit of 5G communication, as it enables operation across widely separated bands and supports multi-operator radio access networks (MORAN). The peak gains observed in the FR1 and FR2 bands are 8.23 dB and 13.14 dB, respectively. The proposed antenna is specifically designed to meet the requirements of the Open RAN compliant shareable 5G small cell radio unit specifications, by offering resonance in n77/n78 and n257/n261 bands, and end-fire radiation characteristics, ensuring sufficient coverage in the indoor and dense urban outdoor environment.

## Introduction

The recent advancements in high-data-demanding applications including 4k streaming platforms, immersive technologies such as virtual reality (VR) headsets, HD content sharing platforms and rapidly increasing smart devices, have necessitated network densification, which require deployment of more small cell access points. In the 5G era, small access points play an important role in fulfilling the gaps of macro-cell coverage, especially in indoor scenarios. Approximately 90% of the overall internet traffic is generated from indoor scenarios, indicating a high demand for efficient and reliable small cell solutions in buildings^1^.

Small cells provide improved coverage and capacity for indoor spaces by employing low-power transmission^2^. However, the traditional small cells are proprietary in terms of both software and hardware components, typically designed and manufactured by a single vendor. Moreover, deployment of a dense network of small cells not only involves a high level of investment in terms of site acquisition (meet-me-room), technology acquisition and management, but also lacks the interoperability that limits the scalability and flexibility of the network^3^. This limitation is addressed through the disaggregation of radio access network (RAN) which is achieved through Open RAN (O-RAN). It involves an open radio unit (O-RU) which can either act as an RF transmission component or it can also perform radio processing functionalities depending on the type of O-RAN implementation. The functions of the traditional baseband unit (BBU) are disaggregated among Open Distributed Unit (O-DU) and Open Central Unit (O-CU)^4^. Other components of the O-RAN include Radio Intelligent Controller (RIC), which performs intelligent network management.

**Fig. 1:**
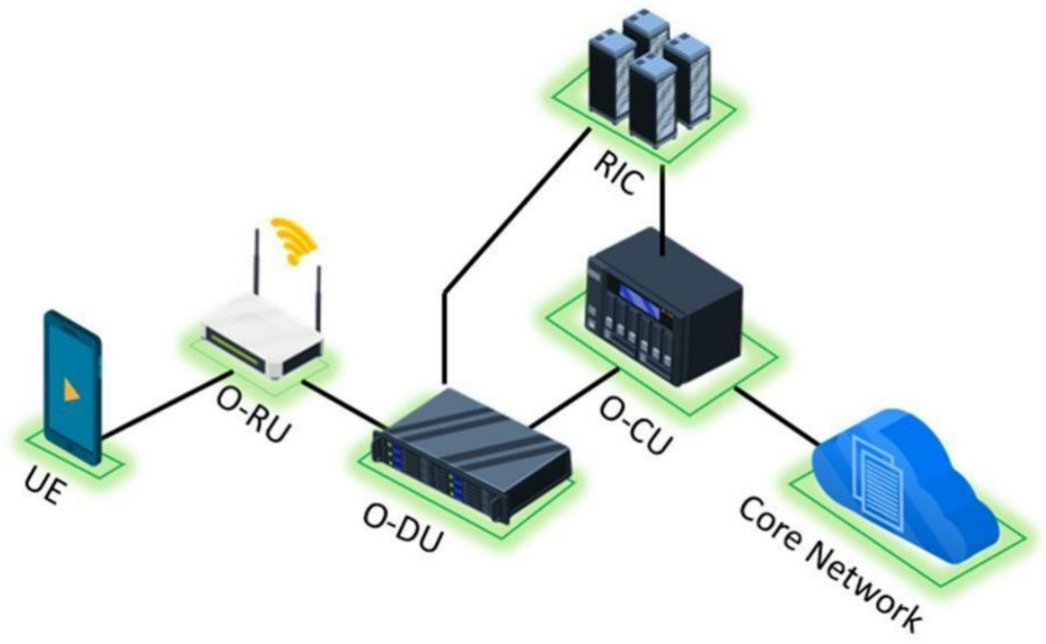
Disaggregated RAN components of the Open RAN Architecture.

**Fig. 2:**
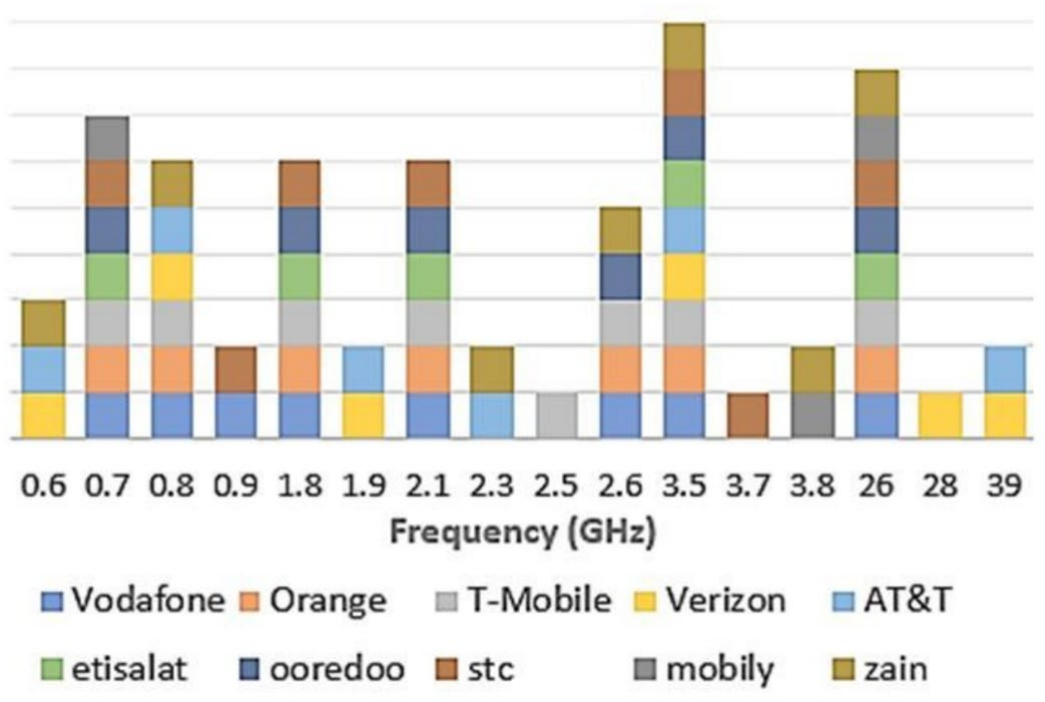
Spectrum utilization by global Mobile Network Operators (MNOs).

A high-level O-RAN architecture is shown in Fig. 1. A shareable 5G small cell solution employing involves a shared O-RU which can be utilized by more than one mobile network operator (MNO)^5,6^. This requires the O-RU to be able to cover large range of frequency bands. More specifically, it is important for an O-RU to be able to have operational frequency in both sub-6 GHz and the millimeter wave (mmWave) bands^7^. The overview of frequency band deployment by different global MNOs is depicted in Fig. 2. It can be observed that the 3.5 GHz and 26 GHz are the most commonly used frequency bands among the MNOs. A survey of spectrum allocation depicts that 5G spectrum ranging from 3 to 4 GHz and 24 to 30 GHz is among the most widely utilized spectrum globally^8^.

The design of an antenna employing multi-band resonance with large frequency ratio (LFR) has recently gained attention due to increasing demand^9–13^. However, these antennas involve conducting antenna elements for mmWave band resonance, which comes with significant shortcomings. The conducting elements suffer from inadequate efficiency, low gains and high conducting losses. Dielectric Resonator Antennas (DRA), on the other hand, not only offer higher efficiency but also provide more design freedom due to their 3-dimensional shapes and choice of different dielectric materials.

A dual-band LFR antenna employing a DRA was reported for the 3.4 GHz and 28 GHz frequency bands^14^. The antenna was designed using cylindrical shaped DRAs arranged in 1 × 4 array formation. The antenna offers impedance bandwidth (IBW) of 190 MHz (3.28–3.47 GHz) and 900 MHz (27.52–28.42 GHz) realizing LFR resonance. The peak gain of the antenna was reported to be 6.16 dBi and 12.3 dBi in the FR1 and FR2 bands, respectively. Despite a promising frequency ratio and peak gain in both bands, the antenna only has a narrow IBW, making it unsuitable for 5G MORAN applications.

Another similar antenna configuration was employed using 2 elements of rectangular DRAs combined with 4 cylindrical DRAs arranged in an array^15^. The antenna is reported to operate at 4.9 GHz and 21.5 GHz having FBW of 30.5% and 21.5% and peak gains of 6.6 dBi and 12 dBi for both low and high frequency bands, respectively. A similar antenna was reported in^16^, which combined an array of DRAs encapsulated a larger DRA for dual-band resonance. This antenna exhibits resonance at two widely separated frequency bands, i.e. 3.35 GHz having a peak gain of 7.8 dBi and 27 GHz having a peak gain of 19.7 dBi. Despite the potentially satisfying radiation characteristics, these 3D printed antennas involve complex 3D designs which require very high precision for fabrication. A relatively simpler antenna design as presented in^17^, which involved a singly-fed shared aperture DRA for dual-band resonance employing LFR characteristics. The antenna offered resonance in 4.44 GHz and 27.92 GHz, having an FBW of 4.1% and 19.8% in the FR1 and FR2 bands, respectively.

A dual-band DRA was designed using 3 different dielectric structures to form a mushroom-shaped structure^18^. The lower part of the DRA was integrated within the substrate, resonated in the mmWave frequency band (at 31.5 GHz). The upper, larger part of the DRA acted as a radiator for the lower frequency band (5.3 GHz) and as a lens for the mmWave band. The FBW in the lower band was reported to be 21% and in the mmWave band to be 26.2%. The peak gains in both bands were observed to be 6.4 dBi and 12.7 dBi. An annular DRA was proposed offering enhanced bandwidth of 52.6% in the FR1 band^19^. The annular DRA was composed of alumina material having dielectric constant of 9.2 having thickness of 3.53 mm and inner air-gap of 7.01 mm. The antenna offered peak gain of 10.15 dBi and circular polarization with axial ratio bandwidth of 1.2 GHz. However, the annular DRA was designed for single band in FR1 range only and lacks in offering dual-band resonance with large frequency ratio characteristics.

In this letter, a 4-element hybrid MIMO antenna is presented that employs a modified cylindrical DRA (CDRA) and a parasitically loaded U-shaped microstrip antenna for a 5G small cell O-RU unit. This work extends our previous research presented in^20^, in which a hybrid DRA was presented. The antenna presented in the previous work consisted of identical antenna elements that were orthogonally placed and excited through ports that were excited via the wave-ports oriented along all 4 edges of the substrate. The peak of the antenna was significantly low, making it limited to indoor applications only. This work presents a novel antenna configuration with enhanced gain and ease of integration with the RF front-end (RFFE) of the radio unit for O-RAN compliant private 5G applications. The antenna was designed to operate in the n78 band in the FR1 regime, and n257, n258, n260 and n261 bands in the FR2 regime, in order to offer enhanced coverage and capacity for shareable small cells for 5G MORAN applications. The U-shaped microstrip radiates in the lower frequency band, whereas it acts as a feeding network for the DRA in the mmWave regime.

## Antenna design process

### MIMO antenna configuration

**Fig. 3:**
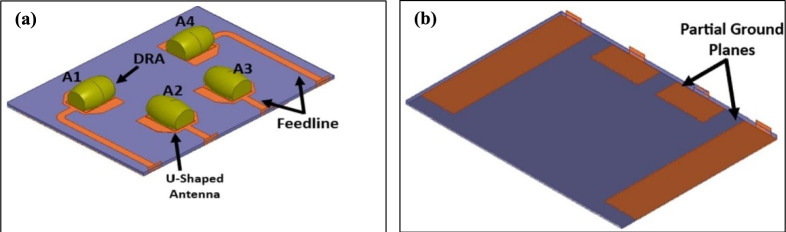
Hybrid MIMO Antenna composed of PB-DRA and Microstrip Antenna **a** top, and **b** bottom view.

The proposed antenna’s geometrical structure is depicted in Fig. 3. Each MIMO element consists of a perturbed barrel shaped DRA (PB-DRA), that is placed over the microstrip antenna. The antenna elements are excited using microstrip feeding network. The microstrip feeds of the elements A1 and A3 are placed in the mid-section of the substrate, whereas A2 and A4 are placed at the edges. A2 and A4 are excited using extended feedline in order to keep the excitations ports on the same edge of the substrate. This is crucial for integration of the antenna with the 5G small cell O-RU’s RFFE unit. The partial ground planes of the elements A2 and A4 are also extended to provide grounding to the feedline as shown in Fig. 3b. Partial grounding structures are designed to ensure omnidirectionality to meet the requirements of indoor 5G coverage^21,22^.

### Design evolution

**Fig. 4:**
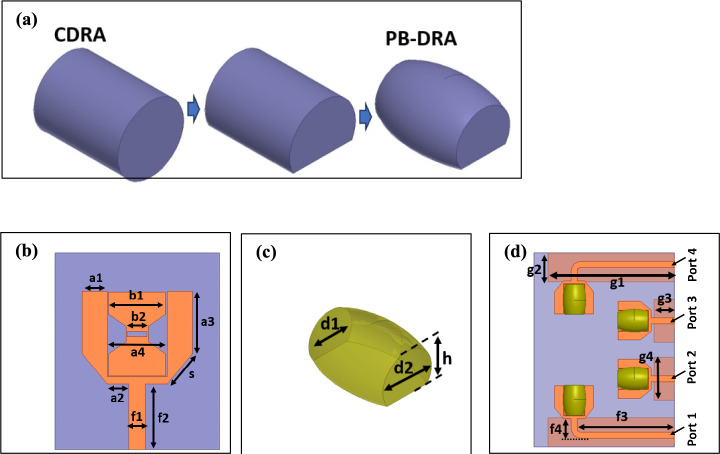
Single Element Design Evolution **a** evolution of DRA design, **b** bow-tie loaded U-shaped antenna design parameters, **c** PB-DRA design parameters and **d** 4-element antenna configuration.

The microstrip antenna elements are designed over an FR-4 substrate having a 10 cm width which is aligned with the size of the O-RU housing. A single element of the proposed MIMO is designed using a U-shaped microstrip radiator which is parasitically loaded with a bow-tie between the arms of the U-structure as shown in Fig. 4b. The dimensions of the antenna are presented in Table 1. A cylindrical DRA is structure is modified by perturbing the structure and converting into a PB-DRA by cutting it into half along its length as shown in Fig. 4a. The transition of DRA configuration from CDRA to PB-DRA is intended to form a hybrid antenna element, that ensures reduced form factor and can be excited through the microstrip structure underneath. The perturbation of the DRA in the initial step leads an asymmetric DRA structure, which is excited in higher order mode to offer resonance in the FR2 range. Furthermore, this modification is intended to reduce the rotational symmetry, which results in the reduction of field confinement and reduced Q-factor. The reduction in Q-factors results in enhanced IBW^23^. Moreover, the DRA results in higher resonant frequency as compared to the CDRA of the same volume due to the reduced dielectric loading^24^. The PB-DRA is composed of material having a dielectric constant of 8 and loss tangent of 0.002, and it is placed on the top of each conducting element. The conducting radiating element is designed to resonate in the sub-6 GHz regime while the PB-DRA is designed for the mmWave band. The conducting radiating element acts as a feeding structure for the PB-DRA in the mmWave regime. Since the resonant frequency of the PB-DRA cannot be analytically computed due to its complex structure which deviates from basic DRA shapes^25,26^. The CDRA of the initial stage of the design process shown in Fig. 4 is considered to approximate the field analysis of the PB-DRA.

**Table 1:** Design parameters of the proposed antenna.

Parameter	Dimension (mm)	Parameter	Dimension (mm)
a1	4.5	f1	3
a2	4	f2	12
a3	11.45	S	7
a4	11	h	7.8
b1	10.4	d1	8.8
b2	3.6	d2	10
g1	65	g4	22
g2	15	f1	3
g3	10.35	f2	12
f3	50	f4	10

The closed form expression (i.e. Eq. 1) for the resonant frequency of the cylindrical DRA given in^27^,

1 $${f}_{r}=\frac{c}{2\pi \sqrt{{\varepsilon }_{r}}}\sqrt{{\left(\frac{{x}_{nm}}{r}\right)}^{2}+{\left(\frac{2\pi }{l}\right)}^{2}}$$

where *r* and *l* are the radius and length of CDRA and *x* represents the root of the first type of Bessel function. The analysis of the excitation mode of the proposed DRA can be carried out by approximating it with hemispherical dielectric structure and analyzing the hybrid mode fields that can be represented as the linear combination of TE and TM-type spherical vector harmonics. The continuity of the tangential fields at the interface between dielectric and air leads to the characteristic matrix equation, which is derived from the source free-Maxwell eigenvalue equation. The matrix equation can be expressed as (i.e. Eq. 2),

2 $$M\left({k}_{o}\right)\left[\genfrac{}{}{0pt}{}{A}{B}\right]=0$$

where the M matrix can be defined with its elements given below,

3 $${M}_{11}=\frac{{{\varphi }{\prime}}_{n}({k}_{d}a)}{{\varphi }_{n}({k}_{d}a)}-\frac{{{{\varepsilon }_{r}\xi }{\prime}}_{n}({k}_{o}a)}{{\xi }_{n}({k}_{o}a)}$$

4 $${M}_{22}=\frac{{{\varphi }{\prime}}_{n}({k}_{d}a)}{{\varphi }_{n}({k}_{d}a)}-\frac{{{\xi }{\prime}}_{n}({k}_{o}a)}{{\varepsilon }_{r}{\xi }_{n}({k}_{o}a)}$$

5 $${M}_{12}=j{\beta }_{n}\frac{{{\varphi }{\prime}}_{n}({k}_{d}a)}{{\varphi }_{n}({k}_{d}a)}-\frac{{{\xi }{\prime}}_{n}({k}_{o}a)}{{\xi }_{n}({k}_{o}a)}$$

6 $${M}_{21}=-j{\beta }_{n}\frac{{{\varphi }{\prime}}_{n}\left({k}_{d}a\right)}{{\varphi }_{n}\left({k}_{d}a\right)}-\frac{{{\xi }{\prime}}_{n}\left({k}_{o}a\right)}{{\xi }_{n}\left({k}_{o}a\right)}$$

**Fig. 5:**
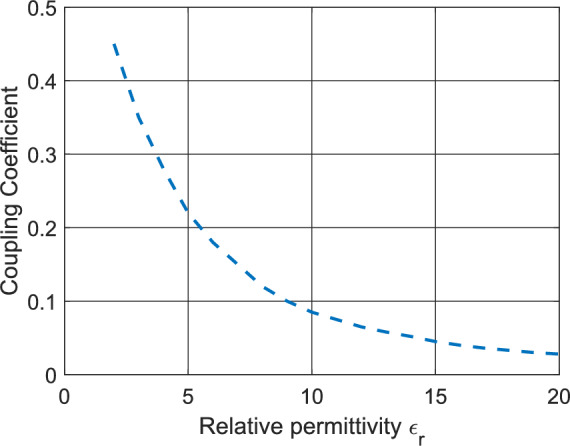
Hybrid coupling coefficient plotted against relative permittivity for DRA operating in HEM_*22δ*_ mode.

Equations (3–6) are Bessel–Hankel function ratios, which determine the interaction between TE and TM modes. Elements *M*_*11*_ and *M*_*22*_ represent tangential field continuity at the boundary of DRA. Off-diagonal elements represent the interaction of TE-TM hybridization, which is scaler multiple of the coupling factor ${\beta }_{n}$. The uncoupled modes are obtained when the ${\beta }_{n}$ approaches zero. Obtaining the determinant of the matrix M leads to,

7 $$\left[\frac{{{\varphi }{\prime}}_{n}\left({k}_{d}a\right)}{{\varphi }_{n}\left({k}_{d}a\right)}-\frac{{{{\varepsilon }_{r}\xi }{\prime}}_{n}\left({k}_{o}a\right)}{{\xi }_{n}\left({k}_{o}a\right)}\right]\left[\frac{{{\varphi }{\prime}}_{n}\left({k}_{d}a\right)}{{\varphi }_{n}\left({k}_{d}a\right)}-\frac{{{\xi }{\prime}}_{n}\left({k}_{o}a\right)}{{\varepsilon }_{r}{\xi }_{n}\left({k}_{o}a\right)}\right]+{\beta }_{n}^{2}\left[\frac{{{\varphi }{\prime}}_{n}({k}_{d}a)}{{\varphi }_{n}({k}_{d}a)}-\frac{{{\xi }{\prime}}_{n}({k}_{o}a)}{{\xi }_{n}({k}_{o}a)}\right]=0$$

where $\varphi$ and $\xi$ are the radial function coefficients and ${\beta }_{n}$ is the mode coupling coefficient. The first two terms in Eq. 7, represent the TE and TM dominant modes, whereas the last term represents the hybridization of modes. The coupling coefficient for varying dielectric constants of a cylindrical DRA can be approximated using a model-overlap perturbation method. Figure 5 shows the profile of the coupling coefficient plotted against the dielectric constant, and it can be observed that dielectric materials with higher relative permittivity led to higher decoupling of modes. On the other hand, smaller dielectric constants lead to poor mode excitation, leading to unstable resonance. Therefore, the choice of material plays a crucial role for an LFR antenna in achieve stable resonance in both bands. This method offers semi-analytical approximation when the close-form solution of the HEM_22δ_ mode does not exit.

The operating mechanism of the proposed antenna is based on frequency-selective excitation of two distinct antenna parts (i.e. a conducting part consisting of bow-tie loaded U-shaped antenna and PB-DRA). The conducting part of the antenna acts as radiator in the sub-6 GHz regime, and the PB-DRA remains a passive dielectric element offering a shift to resonant frequency of the U-shaped structure, which is a deliberate design consideration to achieve resonance in the desired band (n77/n78). Whereas in the mmWave band, the U-shaped conducting structure offers feeding mechanism for the PB-DRA. Resonance in the mmWave band is offered by the excitation of the PB-DRA.

The coupling factor and the Q-factor for HEM_12δ_ mode in a CDRA is expressed in terms of the relative permittivity as,

8 $${\beta }_{n}=(3.27+0.4464x+0.2232{x}^{2}+0.0521{x}^{3}-2.65{e}^{-1.25x(1+4.7x)})\frac{r}{\sqrt{{\varepsilon }_{r}}}$$

9 $$Q= {\varepsilon }_{r}^{2}\left(0.068-0.0388x+0.0064{x}^{2}+0.0007{e}^{x(37.59-63.8x)}\right)$$

where *x* is expressed in terms of the radius and length of the CDRA as x = r/2* l* ^27^. Equations 8 and 9 indicate the DRA having a smaller dielectric constant would lead to dominance of hybrid modes and a smaller Q-factor, since the asymmetric structure reduces field confinement and increases the ratio of effective radiating aperture to the stored energy. Therefore, choice of material plays very important role when designing higher order mode DRA. DRAs having higher relative permittivity offer less hybridization as the coupling factor is less and the coupling term in Eq. 7 has lesser impact. However, it also reduces the bandwidth due to a higher Q-factor as evident from Eq. 9. The relative permittivity of the proposed DRA is 8, which makes it adequate to acquire significant bandwidth in the higher order mode.

The parasitic bow-tie structure with a center slot having a length of 3.6 mm is designed for the enhancement of the gain of the U-shaped antenna in the FR1 band. For this length, the resonant frequency of the bow-tie was evaluated using the analytical method presented in^28^. The evaluated resonant frequency was found to be 6.3 GHz, which is specific for a bow-tie patch resonating in the fundamental mode (TM_01_). The parasitic excitation leads to reactive coupling which effectively increases the electrical length of the structure. Moreover, the passive loading of the PB-DRA in the sub-6 GHz acts as dielectric superstrate, resulting in shifting the resonance to a lower frequency^29^. The passive loading of the DRA results in effective permittivity of ~ 4.7 resulting in a shift of resonant frequency from 6.3 GHz to 2.9 GHz.

**Fig. 6:**
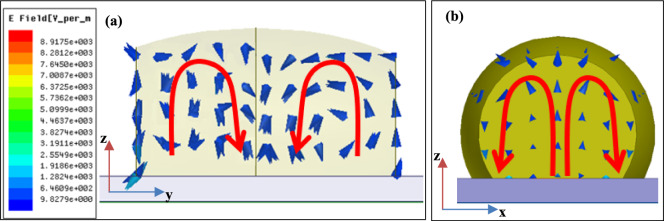
Electric Field distribution in the PB-DRA depicting HEM_22δ_ resonant mode **a** xz-plane crossection and **b** yz-plane side view.

**Fig. 7:**
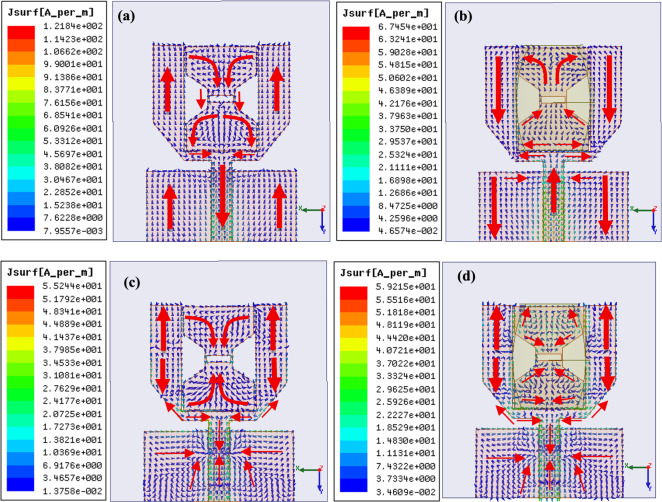
The simulated current distribution **a** in the conducting antenna at 3.85 GHz, **b** PB-DRA integrated antenna element at 3.8 GHz **c** in the conducting antenna at 26 GHz and **d** PB-DRA integrated antenna element at 28 GHz.

Figure 6 depicts the electric field distribution in the PB-DRA at the FR2 frequency band (25 GHz). It is observed that the cDRA has a hybrid higher order mode (HEM_22δ_) which resembles the quasi-HEM_21δ_ in the rDRAs that produces an omni-directional pattern^30^. The observed mode in the PB-DRA is higher order hybrid mode, which is caused by the perturbation in the canonical shaped CDRA. The dual-lobe E-field pattern is observed in the field distribution indicating second order field variations, which relates to HEM_22δ_ mode. However, the E-field in the PB-DRA at FR1 band remains non-modal due to a smaller electrical size of DRA. the Fig. 7 represents the simulated surface current distribution in the conducting element at 3.85 GHz and 26 GHz. It is observed through the surface current distribution that the PB-DRA contributes by increasing the effective aperture of the antenna in both frequency bands. The inclusion of the PB-DRA not only contributes through its own radiation in the mmWave band but also enhances the current distribution in the microstrip radiator.

### Results and analysis

**Fig. 8:**
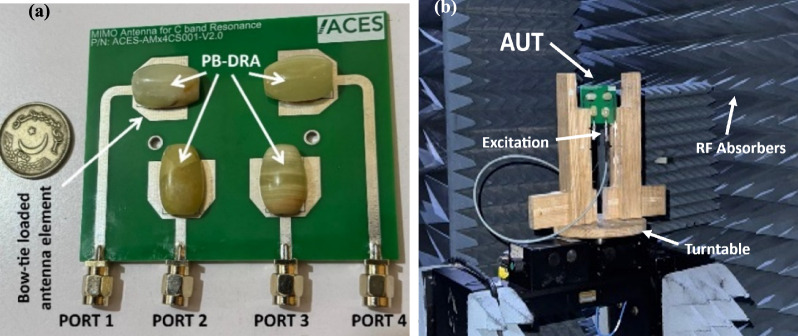
Prototype of Hybrid MIMO Antenna.

The proposed modified CDRA was fabricated through the abrasive water-jet cutting procedure whereas the Lasor Direct Imaging (LDI) was used for microstrip radiator etching on the FR4 substrate. The water-jet cutting offers a tolerance of ± 0.5–1 mm, which is adequately precise for DRA fabrication. The fabricated prototype of the antenna is shown in Fig. 8a and b depicts the measurement setup of the antenna in the anechoic chamber.

**Fig. 9:**
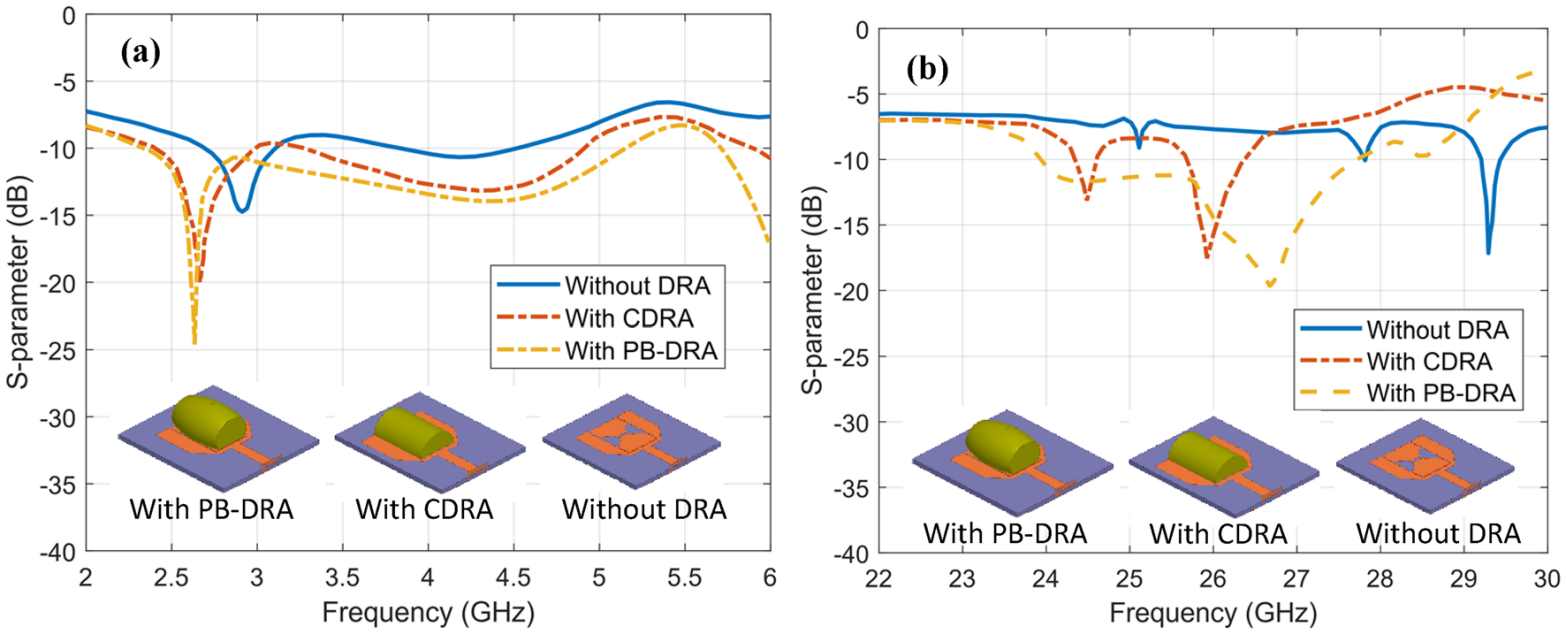
Simulated results of s-parameter for single antenne element without DRA integration, with CDRA and with PB-DRA at **a** FR1 band and **b** FR2 band.

**Fig. 10:**
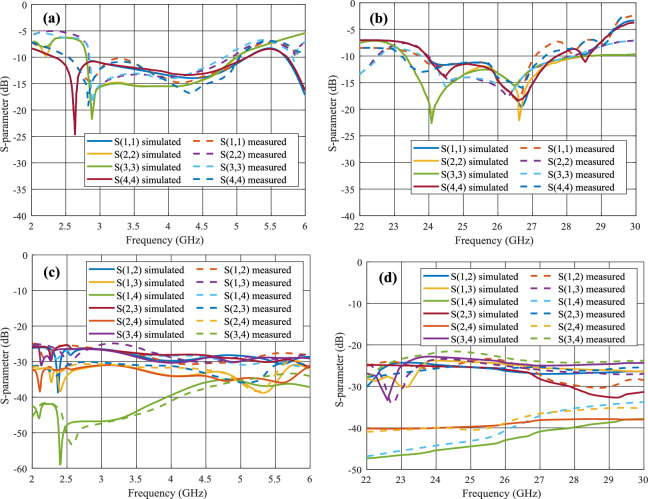
Simulated and measured results depicting **a**, **b** reflection coefficient and **c**, **d** mutual coupling of the 4-element MIMO Antenna.

S-parameter profiles shown in Fig. 9 depict the effect of CDRA and PB-DRA integration on the U-shaped microstrip antenna. The reflection coefficient in the sub-6 GHz band appears to have improved coupling, depicted by deeper minima in the S-parameter profile as depicted in Fig. 9a. Similarly, in the mmWave band, the antenna without the PB-DRA shows no resonance at 26 GHz and one dominant dip at 29 GHz. The inclusion of CDRA and PB-DRA introduces s-parameter minima between 24 and 27 GHz which correspond to DRA’s higher order resonance modes. Simulated and measured results shown in Fig. 10 represent good agreement. The mutual coupling of the MIMO antenna elements is observed to be below – 25 dB over the entire range of both operational frequency bands as shown in Fig. 10b, d. The proposed hybrid antenna obtains low mutual coupling by achieving field distribution and modal orientation through hybrid excitation, dielectric confinement, and reactive loading. These mechanisms in combination break up the coupling paths (surface waves and co-polarized near-field overlap), enabling enhanced isolation to be preserved over both bands. The complete analysis of the measured s-parameter results validates the dual-band resonance with LFR having a frequency ratio 6.92. In the sub-6 GHz frequency range, the antenna offers IBW ranging from 2.84 GHz to 4.86 GHz. Fractional bandwidth is observed to be 52.47% in the FR1 band, and it covers the entire n77 and n78 5G bands, which are most widely used in numerous 5G deployment use cases. In the mmWave frequency range, the proposed PB-DRA covers the IBW of 24 GHz to 29.3 GHz, having fractional bandwidth of 19.9%. This range covers the entire n257, n258, n260 and n261 of the 5G NR FR2 which makes the proposed antenna a potential candidate for the 5G small cell deployment in dense urban hotspots, crowded indoor scenarios such as sports arenas and private 5G in-building solutions.

**Fig. 11:**
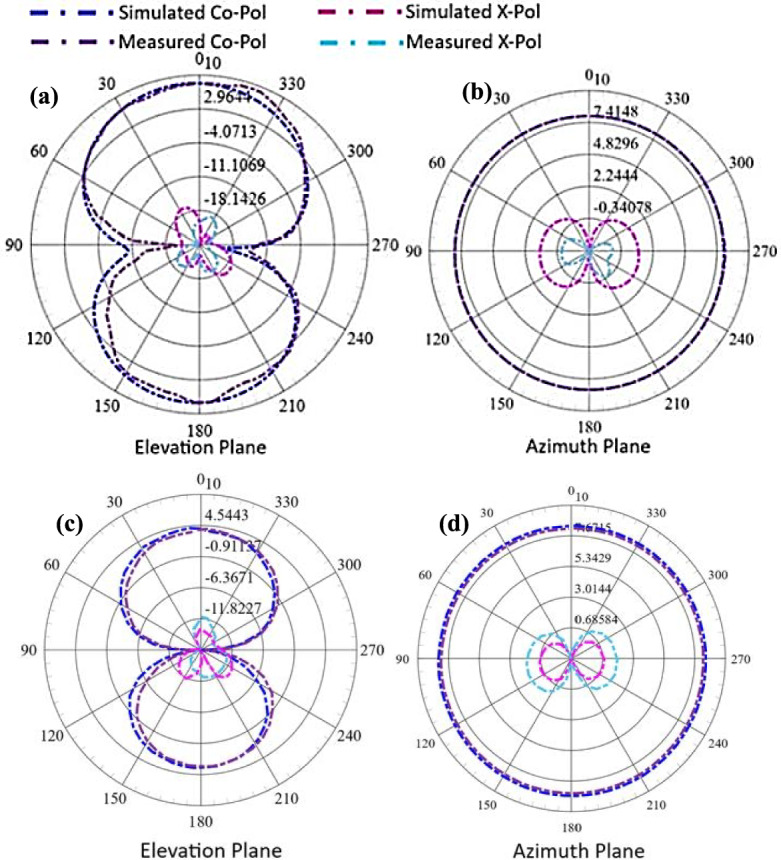
Radiation patterns in elevation and azimuth planes **a**, **b** at 3 GHz and **c**, **d** at 4 GHz.

**Fig. 12:**
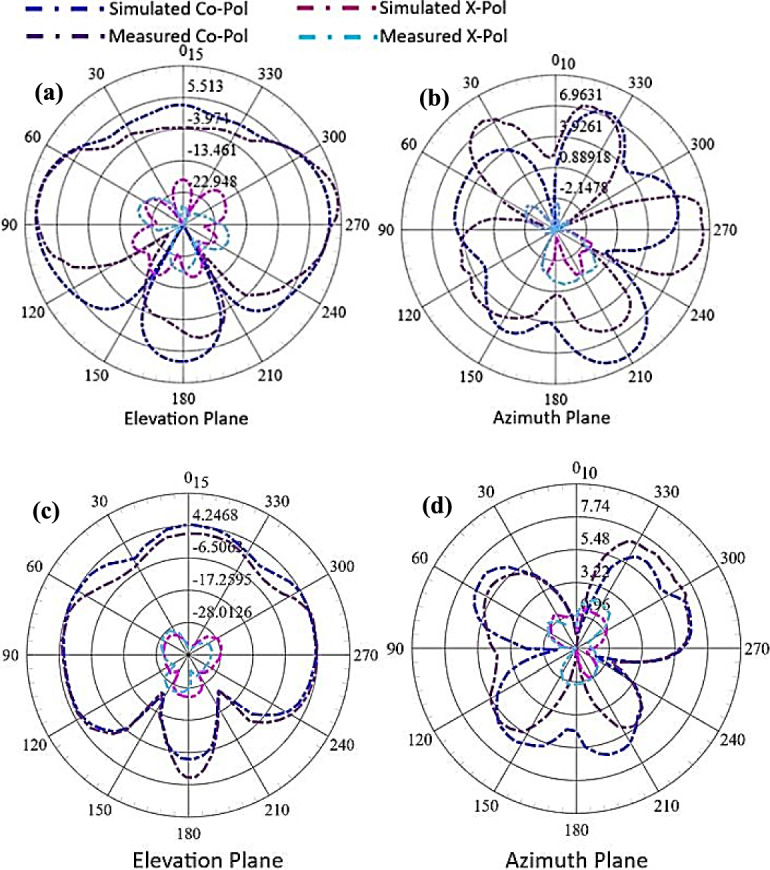
Radiation patterns in elevation and azimuth planes **a**, **b** at 24 GHz and **c**, **d** at 26 GHz.

The far-field radiation characteristics at two different resonance frequencies in each frequency regime (i.e. FR1 and FR2) are presented in Figs. 11 and 12 showing a broadside radiation pattern in the FR1 range and an end-fire radiation pattern in the FR 2 range. The FR1 range radiations shown in Fig. 11 depict a gain of 8.23 dBi with the main-lobe in the broadside direction depicting the microstrip radiator’s TM10-like resonance. Symmetry of the current distribution in both arms of the planar U-shaped structure shown in Fig. 12 results in the E-field which is dominant in the broadside direction. The radiation patterns depict a half-power beamwidth (HPBW) of 81.64° and a minor cross-polarization radiation of – 6.01 dBi which lies under the acceptable range.

The radiation pattern for the FR2 range shown in Fig. 12 represents an end-fire characteristic with the gain of 13.14 dBi. The HEM_22δ_ mode of the proposed PB-DRA offers dominant longitudinal E-field components that concentrate radiation towards the DRA’s ends, whereas the U-shaped microstrip’s arms serve as coupling feeders, phase-synchronizing fields to reinforce end-fire emission, resulting in a quite high gain in the FR2 range. The end-fire radiation pattern at the FR2 range is required to meet the requirements of the 5G small cell RU, which is aimed to be used as an indoor access point. Therefore, the end-fire at the FR2 range ensures strong line-of-sight (LOS) coverage along the axis of deployment. The HPBW in the FR2 range is 47.48° and cross polarization of – 5.7 dBi.

**Fig. 13:**
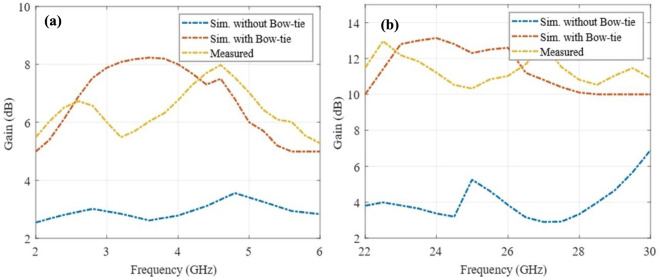
Measured and simulated gain profile depicting gain enhancement in **a** FR1 band, and **b** FR2 band.

The gain profiles in the FR1 and FR2 frequency regimes of the composite MIMO element are presented in Fig. 13. It can be observed that the gain enhancement of over 4 dBi is achieved through the integration of a parasitically excited bow-tie shaped patch with a rectangular slot between the arms of the U-shaped radiator. The field distribution in the bow-tie structure depicted in Fig. 7d, matches the hybrid field symmetry of the PB-DRA resulting in the gain enhancement in FR2 frequency rage as depicted in Fig. 13b.

The proposed antenna was integrated with the 5G small cell radio unit for testing the proof-of-concept of a private 5G network in a real-world environment. The testing setup involved Open 5GS core and srsRAN for the deployment of an Open RAN compliant private 5G testbed. Reference Signal Received Power (RSRP) measurement was carried out through the indoor environment and a minimum of – 75 dB RSRP was observed in a floor-plan covering approximately 240*m*^2^ of coverage area.

**Fig. 14:**
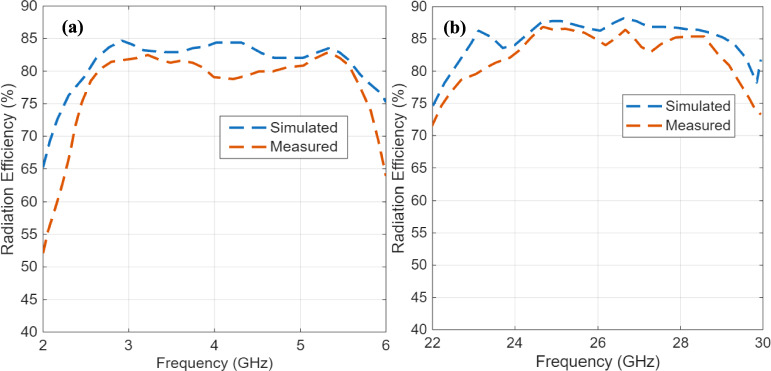
Measured and simulated radiation efficiency profiles in **a** FR1 band, and **b** FR2 band.

Table 2 shows a comparative analysis of the designed hybrid antenna with other LFR antennas in the recent literature. It can be clearly seen that the proposed structure outperforms other current configurations in both bands to a considerable extent. Under the sub-6 GHz (FR1) frequency, the antenna has an extremely high fractional bandwidth (FBW) of 52.6%, 1.7 × larger than the largest bandwidth reported in similar designs^7^, and nearly 10 × larger than array-type DRAs^5^ and composite DRAs^9^. This improvement is due to the geometric perturbation of the cylindrical DRA into the perturbed-barrel form, which reduces the quality factor by enhancing radiation leakage and impedance matching over a broader spectrum. The parasitically coupled bow-tie patch also increases the effective aperture, leading to a measured broadside gain of 8.23 dBi that represents a significant improvement over the 6–7 dBi range of typical CDRA and MIMO-DRA designs. Figure 14 depicts the measured and simulated radiation efficiency profiles of the proposed antenna.

**Table 2:** Summary of the performance of related antennas and the proposed antenna.

Reference	Antenna Type	FR1%BW	Gain (dB)	FR2%BW	Gain (dB)	Height of antenna (mm)	Area profile (mm^2^)
^14^	Array	5.63	6.16	3.22	12.3	7.5	50 × 50
^15^	MIMO	30.5	6.6	21.5	12	9.25	60 × 60
^16^	Encapsulated DRA	20.2	7.8	28.7	7.8	20.75	30 × 30
^17^	Composite DRA	4.1	4.3	19.8	6.8	20	150 × 150
^18^	Mushroom Shaped DRA	21	6.4	26.2	12.7	16.44	75 × 42
^31^	Dipole	41.38	9	11	9.7	3.11	400 × 400
^32^	Patch + metasurface	7.52	6.2	14.55	19.3	10	100
^33^	Patch + metasurface	23.45	10.44	9.76	14.6	7.34	92 × 92
^34^	Patch + PRS	3.7	4.85	9.71	22.5	24	100 × 100
This Work	Hybrid DRA	52.6	8.23	19.9	13.14	7.8	100 × 73

The peak radiation efficiency of the proposed antenna is observed to be 84.6% and 87.5% in the FR1 and FR2 bands, respectively. In the mmWave (FR2) band, the designed antenna still offers a wide 19.9% FBW and reaches a peak gain of 13.14 dBi, outperforming all the benchmark designs in Table 2. The reason is mainly attributed to the excitation of the HEM_22δ_ mode in the PB-DRA, whose longitudinal field components enhance the end-fire radiation produced by the U-shaped microstrip feed. The resultant hybrid coupling between the microstrip and dielectric resonator domains is one that provides an extremely directive end-fire pattern without sacrificing impedance bandwidth. Higher-order mode excitation is a deliberate design selection based on the hybrid integration approach. To induce resonance within the mmWave band through the same port, the PB-DRA is designed to couple effectively with the fringing fields of the U-shaped radiator, which tend to excite the higher-order hybrid modes.

#### Diversity parameters

MIMO antenna performance was validated by evaluating diversity parameters including the envelope correlation coefficient (ECC), the diversity gain (DG), total active reflection coefficient (TARC) and mean effective gain (MEG). ECC is the measure of isolation between multiple communication channels. It can be evaluated either by considering the s-parameters of each MIMO element, or by using the field radiation-patterns^35^. The method involving s-parameters is typically considered less accurate, since it only incorporates the port isolation due to the geometry of the antenna. On the other hand, the ECC evaluation using radiation-patterns involves mutual coupling caused by radiations of nearby MIMO elements. ECC of the proposed antenna was evaluated using the field analysis method^35^. The ECC is found to be below 0.01 over the operational bands, which ensures strong isolation and an uncorrelated radiation pattern. The ECC value is small because of the use of partial and discrete ground planes and asymmetric element placement. Moreover, the DG was evaluated using the full-wave simulator. It was observed to be ~ 9.8 dB, which signifies potential MIMO performance.

**Fig. 15:**
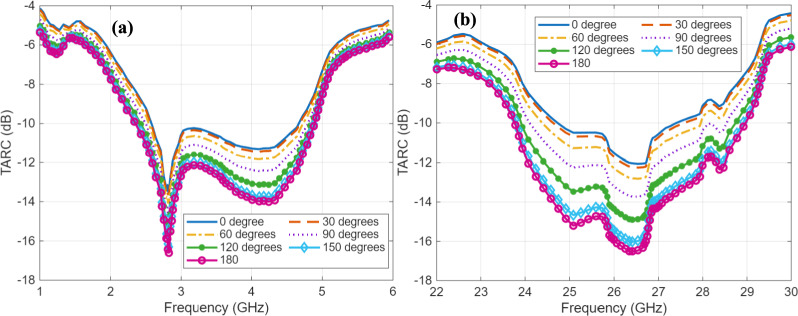
TARC profiles in **a** FR1 band, and **b** FR2 band.

TARC of a MIMO antenna is the evaluated using the square root of ratios of incident power to the reflected power^36^. Evaluation of TARC is essential for the performance analysis of a MIMO antenna, since the s-parameters do not offer insight into simultaneous excitation of all MIMO antenna elements. Furthermore, TARC also takes into account the phase progression in the incident waves. The TARC profiles in the FR1 band and FR2 band are depicted in Fig. 15. The phase progression represents difference of phase among adjacent excitations which goes from 0 degrees to the 180 degrees. It is observed that the TARC in the entire operational frequency range of 2.84–4.86 GHz in the FR1 band, and 24–29.3 GHz in the FR2 band is less than – 9 dB. This range of TARC is has been reported to provide good efficiency and MIMO performance^37^. MEG is of the proposed MIMO antenna is evaluated using the 3D radiation patterns as expressed in^38^. It is expressed as the ratio of received power averaged over all MIMO ports to the average incident power. The MEG of the proposed antenna was observed to be –5.3 dB ensuring balanced and efficient contribution of each MIMO element.

## Conclusion

A hybrid MIMO antenna consisting of 4 elements is proposed in this article. Each antenna element is designed using a U-shaped microstrip radiator, which is parasitically loaded with a bow-tie shaped patch in the middle. Moreover, each element consists of a perturbed barrel-shaped DRA having a permittivity of 8, which is designed for the FR2 frequency range. The conducting part of each element is designed to resonate in the FR1 range giving the antenna a dual-band resonance with LFR characteristics. The antenna offers excellent radiation characteristics, having impedance bandwidth of 2.02 GHz ranging from 2.84 GHz to 4.86 GHz in the FR1 spectrum. Whereas in the FR2 band, the PB-DRA resonates at 26.65 GHz having an IBW of 5.3 GHz ranging from 24 GHz to 29.3 GHz. The bandwidths observed in the FR1 and FR2 bands cover the entire n77, n78, n257, n258, n260 and n261 bands of the 5G NR, making the antenna a suitable candidate for 5G small cell radio units. The future work of this research can be carried out to introduce frequency reconfigurability for dynamic spectrum MORAN coverage.

## Data Availability

The datasets used and analyzed during the current study are available from the corresponding author upon reasonable request.
